# Redo Pulmonary Vein Isolation After Index Cryoballoon Ablation: Cryoballoon Versus Radiofrequency Redo Ablation for Recurrent Atrial Fibrillation

**DOI:** 10.3390/jcm15187325

**Published:** 2026-09-21

**Authors:** Wasim Schehab, Markus Schlömicher, Assem Aweimer, Gresa Kciku, Snehasis Pradhan, Hans J. Trappe, Justus Strauch, Nazha Hamdani, Ibrahim El-Battrawy

**Affiliations:** 1 Medical Faculty, Institute of Physiology, Department of Cellular and Translational Physiology, Ruhr University Bochum, 44801 Bochum, Germany; d_schehab@hotmail.com (W.S.);; 2 Medical Faculty, Institut für Forschung und Lehre (IFL), Molecular and Experimental Cardiology, Ruhr University Bochum,44801 Bochum, Germany; 3 Department of Cardiology and Rhythmology, Ludmillenstift, Academic Teaching Hospital, 49716 Meppen, Germany; 4 Medical Faculty, Department of Cardiac Surgery, Bergmannsheil University Medical Center, Ruhr University Bochum, 44789 Bochum, Germany; 5 Marienhospital Herne, University Medical Center Bochum, Ruhr University Bochum, 44625 Herne, Germany; 6 Medical Faculty, Department of Cardiology, St. Josef-Hospital, UK RUB, Ruhr University Bochum, 44791 Bochum, Germany; 7 Department of Physiology, Cardiovascular Research Institute Maastricht, Maastricht University, 6229 ER Maastricht, The Netherlands; 8 Department of Cardiology and Rhythmology, Bergmannsheil University Medical Center, Ruhr University Bochum, 44789 Bochum, Germany

**Keywords:** atrial fibrillation, redo ablation, cryoballoon, radiofrequency, pulmonary vein reconnection

## Abstract

**Background:** Data guiding the choice of energy source for redo pulmonary vein isolation (PVI) after index cryoballoon (CB) PVI are limited. We compared procedural findings, pulmonary vein (PV) reconnection patterns, and reported clinical outcomes after CB-redo versus radiofrequency (RF)-redo ablation in patients with recurrent atrial fibrillation (AF) following an index CB-PVI. **Methods:** We included a retrospective single-center cohort of 61 consecutive patients with symptomatic recurrent AF after prior CB-PVI who underwent redo ablation between 2019 and 2020 (CB redo, n = 32 vs. RF redo, n = 29). All patients had an index CB PVI (28 mm balloon). The primary reported outcome was freedom from atrial tachyarrhythmia after the blanking period. **Results:** Mean age was 66 ± 10 years; 41 (67%) were men. Time from index procedure to redo: CB redo 323 ± 72 days vs. RF redo 238 ± 48 days (*p* < 0.001). All patients had ≥1 PV at redo. Mean reconnected PVs per patient: CB 2.1 ± 1.1 vs. RF 1.7 ± 0.6 (*p* = 0.08). Right inferior PV reconnection was more frequent after CB redo (69% vs. 41%; *p* = 0.03). Early recurrence during the 90-day blanking period was CB 8/32 (25.0%) vs. RF 9/29 (31.0%) (*p* = 0.54). At 12 months, recurrence was CB 7/32 (21.9%) vs. RF 6/29 (20.7%) (unadjusted *p* = 0.75). Kaplan–Meier log-rank *p* = 0.88. Adjusted Cox HR (CB vs. RF) = 1.05 (95% CI 0.40–2.76; *p* = 0.92). Total outcome events = 13. No major periprocedural complications were observed. **Conclusions:** No statistically significant difference in arrhythmia recurrence between CB redo and RF redo strategies was detected. However, the small sample size, limited event count, non-random treatment selection, and wide statistical uncertainty preclude conclusions regarding equivalence.

## 1. Introduction

Atrial fibrillation (AF) is the most common supraventricular arrhythmia in the industrialized population [1]. Its incidence in Western countries is high and continues to rise, resulting in an increasing clinical and health economic burden [2,3]. Several cardiovascular risk factors and comorbidities have consistently been associated with the occurrence of AF in the general population [4]. Advanced age, male sex, long-standing hypertension, and obesity are strong predictors of arrhythmia development. In addition, cardiovascular diseases, particularly heart failure, valvular heart disease, and prior myocardial infarction, are associated with a significantly increased risk of AF [5].

Catheter ablation has become an established treatment option for symptomatic AF; however, arrhythmia recurrence remains a relevant clinical problem. Although the highest recurrence risk occurs during the blanking period, late recurrences have been reported in approximately 20–40% of patients. Pulmonary vein isolation (PVI) is a safe and effective cornerstone of AF ablation. Compared with radiofrequency (RF) ablation, cryoballoon (CB) ablation has been associated with shorter fluoroscopy and total procedure times during index PVI procedures [6], while overall clinical outcomes and complication rates appear comparable [7].

However, data regarding the optimal energy source for redo procedures after failed initial cryoballoon ablation remain limited. RF ablation does not require pulmonary vein (PV) occlusion and offers high catheter flexibility with precise tissue contact, allowing tailored lesion delivery. In contrast, CB ablation enables circumferential antral lesions in a single application. One study comparing RF and CB in redo procedures reported a one-year freedom from AF without antiarrhythmic drugs of 58% in RF-treated patients versus 43% in CB-treated patients, although this difference did not reach statistical significance (*p* = 0.06) [8].

In this single-center study, we compared baseline characteristics, procedural parameters, clinical outcomes, and AF recurrence rates between patients undergoing redo pulmonary vein isolation (PVI) with the cryoballoon ablation technique versus radiofrequency ablation after an initial cryoballoon PVI. 

## 2. Methods

### 2.1. Study Design and Population

We conducted a retrospective cohort study including consecutive symptomatic adult patients who underwent redo PVI for recurrent AF after an index cryoballoon PVI at Marien Hospital Herne between January 2019 and December 2020. Inclusion criteria were age ≥ 18 years, prior index cryoballoon ablation with documented AF recurrence (symptomatic and/or ECG- or Holter-documented), and redo PVI performed using either CB or RF.

Exclusion criteria included non-pulmonary vein trigger ablation during the index procedure, left atrial appendage isolation at index ablation, prior surgical AF ablation, or incomplete procedural documentation. Based on local regulations and the pseudo-randomized retrospective collection of data, no ethical approval was required at the time of the study.

### 2.2. Energy Selection and Procedural Strategy

The choice of energy source (CB vs. RF) was determined by the operator and guided by a predefined local algorithm integrating anatomical, electrophysiological, and procedural factors.

The algorithm followed a stepwise decision-making approach:

(1) Anatomical assessment:

Pulmonary vein anatomy was evaluated using pre-procedural imaging (TEE and/or CT/MRI) as well as intra-procedural three-dimensional (3D) mapping. Cryoballoon ablation was preferred in patients with favorable anatomy (four separate pulmonary veins, absence of a common ostium, pulmonary vein diameter approximately 10–20 mm, and no relevant left atrial enlargement), whereas RF ablation was favored in cases of complex anatomy (e.g., common ostia, dilated pulmonary veins, or enlarged left atrium).

(2) Reconnection pattern (redo procedures):

In redo procedures, the pattern of pulmonary vein reconnection was considered. In cases of multiple or diffuse reconnections, a CB approach was preferred, whereas focal or segmental reconnections were treated using RF to allow targeted lesion delivery.

(3) Requirement for additional lesion sets:

If additional substrate modification was required (e.g., posterior wall isolation or linear lesions), RF ablation was preferred due to its superior flexibility and spatial precision.

(4) Procedural considerations:

Procedural aspects were also taken into account. In particular, CB was favored in cases where a shorter procedure duration was anticipated.

## 3. Procedures

### 3.1. Pre-Procedural Management

Transesophageal echocardiography was performed within 24 h before the procedure to exclude left atrial thrombus. Periprocedural anticoagulation followed contemporary guidelines. Oral anticoagulation was continued until the procedure and was paused only on the morning of the procedure, and intravenous heparin was administered during left atrial access to maintain an activated clotting time (ACT) between 300 and 350 s. After the procedure, therapeutic oral anticoagulation was continued. In all patients, anticoagulation was continued permanently (i.e., until the last follow-up).

### 3.2. Sedation and Analgesia

CB procedures were performed under conscious sedation using intravenous midazolam (5 mg) and fentanyl (50 µg). RF procedures were conducted under deeper sedation with continuous propofol infusion and additional fentanyl boluses (50 µg) as required based on sedation depth and patient tolerance.

**Figure 1:**
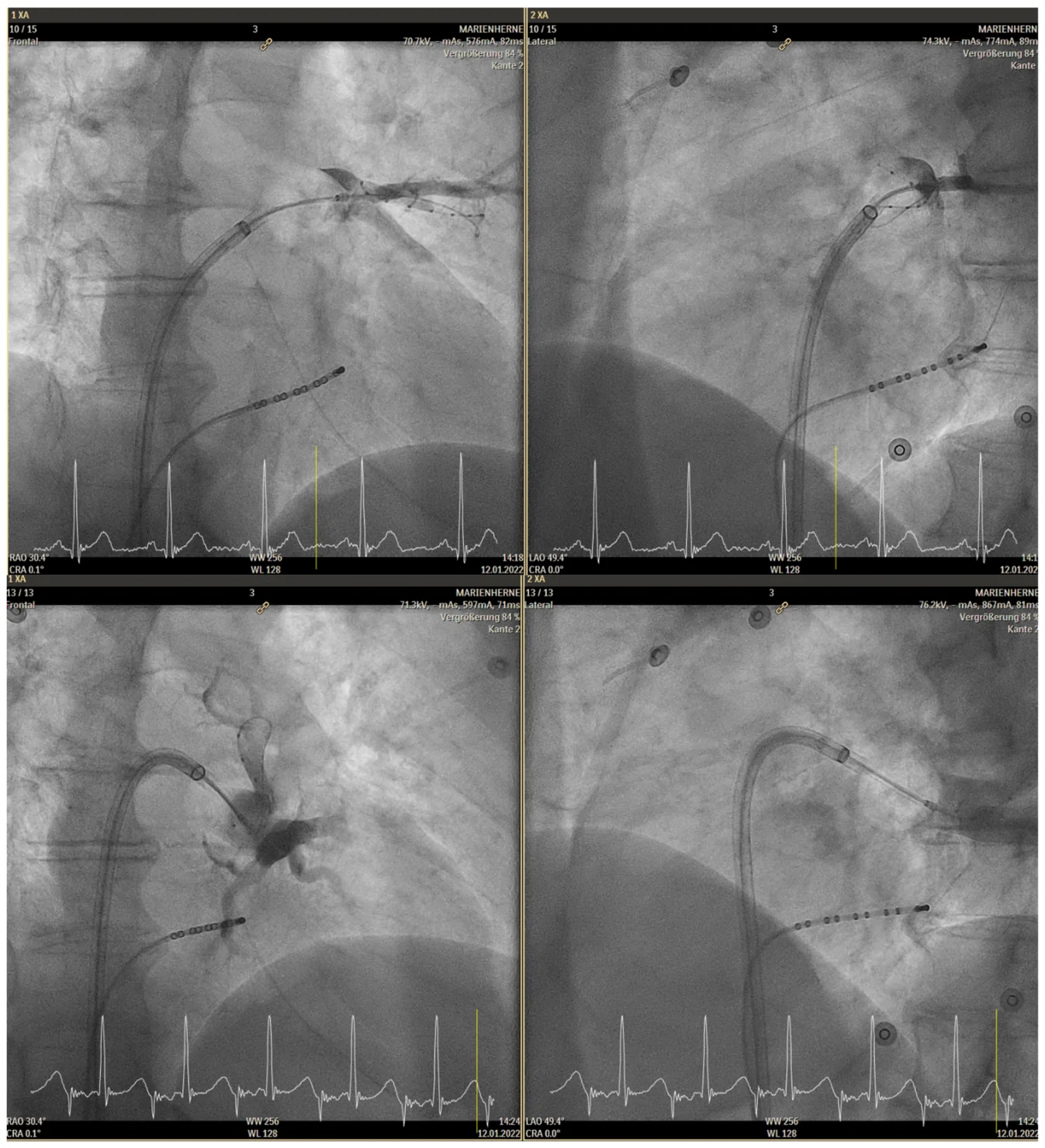
Cryoballoon positioning during ablation in the left superior pulmonary vein (LSPV, upper panels) and left inferior pulmonary vein (LIPV, lower panels) in RAO 30° (left) and LAO 50° (right) projections using the Achieve circumferential catheter. While the balloon may appear slightly oval-shaped in the presented fluoroscopic projection, this does not necessarily indicate deep seating within the pulmonary vein. Balloon shape on fluoroscopy is highly dependent on projection angle, anatomical orientation of the pulmonary vein ostium, and local compliance of the atrial tissue.

Cryoballoon ablation procedure: CB for antral pulmonary vein isolation was performed using a double-walled balloon catheter into which nitrous oxide (N_2_O) was delivered. Two balloon sizes (23 mm and 28 mm) were available; however, only the 28 mm balloon was used in this study (Figure 1). After transseptal puncture via a 9-French sheath, a guidewire was advanced into the left superior pulmonary vein, and a 12-French deflectable sheath (Cryoflex^®^) was positioned in the left atrium.

The cryoballoon catheter (Arctic Front Advance™, Medtronic, Puerto Rico, USA) was advanced over the guidewire to the target PV ostium. The catheter contained a central lumen for guidewire placement and contrast injection. An eight-pole circular mapping catheter (Achieve^®^, Medtronic) was used to assess PV potentials and tissue contact. Adequate balloon-to-tissue contact was confirmed by contrast injection, with complete occlusion inferred when no contrast reflux into the left atrium was observed.

Following confirmation of PV occlusion, N_2_O was introduced into the balloon, resulting in cooling to approximately −50 °C to −60 °C and creation of a circumferential lesion at the PV ostium. Any PV demonstrating residual conduction gaps was re-ablated for 120 s, with extension to 240 s if initial isolation time exceeded 90 s. Oesophageal temperature was continuously monitored, and ablation was terminated if the temperature dropped below 15 °C. During right-sided PV ablation, continuous phrenic nerve pacing via a catheter positioned in the superior vena cava was performed; ablation was immediately terminated in case of diaphragmatic weakening.

After completion of all PV ablations, a 20 min waiting period was observed, followed by reassessment of PV isolation using the Achieve^®^ catheter. Additional ablation was performed if isolation was incomplete.

In our case, the cryoballoon was positioned at the antral level, as confirmed by contrast injection demonstrating adequate pulmonary vein occlusion without distal contrast leakage. Furthermore, the Achieve catheter remained in a stable position within the vein, supporting correct ostial/antral positioning rather than deep intubation.

It is important to note that a perfectly spherical appearance is not mandatory for effective and safe ablation, particularly in anatomies with elliptical ostia or variable angulation. Mild deformation of the balloon contour can occur without compromising correct positioning.

Radiofrequency catheter ablation procedure: Patients were either in spontaneous sinus rhythm or underwent electrical cardioversion before the procedure. Following transseptal puncture, an initial bolus of 10,000 IU heparin was administered to achieve an ACT target of up to 350 s. Two venous sheaths (7F and SL1 9F) were introduced via the femoral vein, and a 10-pole catheter was placed in the proximal coronary sinus.

**Figure 2:**
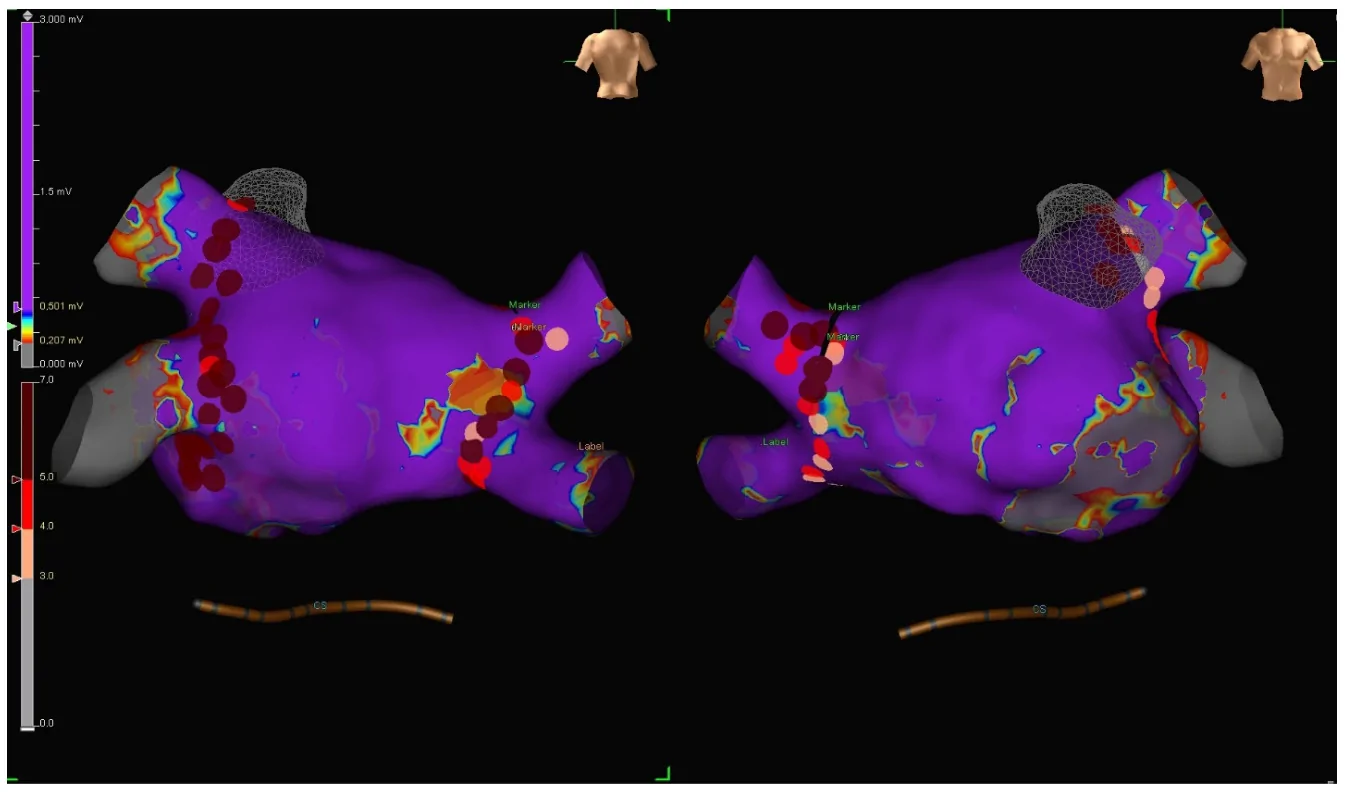
Three-dimensional mapping using the HD Grid catheter with reconstruction of the left atrium using the EnSite Precision system, followed by point-by-point circumferential isolation of all four pulmonary veins. CS = coronary sinus.

Twelve-lead ECGs and intracardiac electrograms were recorded using a multichannel system (Bard Labsystem, Boston Scientific Corporation, Marlborough, MA, USA). After transseptal access with the SL1 sheath, a guidewire was placed in the left superior pulmonary vein, and a deflectable sheath (Agilis^®^, Abbott, St. Paul, MN, USA) was positioned in the left atrium. A high-density mapping catheter (Advisor™ HD Grid Mapping Catheter, Sensor Enabled™, Abbott, St. Paul, MN, USA) was advanced to perform left atrial voltage mapping using the EnSite Precision or Velocity system (Figure 2). Low-voltage areas were defined as bipolar electrogram amplitudes < 0.5 mV during sinus rhythm.

A second transseptal puncture was then performed, and an ablation catheter (TactiCath™, Abbott, St. Paul, MN, USA) was introduced. Catheters were exchanged between sheaths to optimize mapping and ablation. If two PVs on the same side were not isolated, circumferential RF ablation was performed, and entrance and exit blocks were confirmed in each PV.

### 3.3. Periprocedural Care and Follow-Up

Procedures were performed under conscious sedation or general anesthesia at the operator’s discretion. Anticoagulation, oesophageal temperature monitoring, and phrenic nerve pacing were routinely employed. Post-procedural care included telemetry monitoring for at least 24 h and transthoracic echocardiography before discharge.

### 3.4. Follow-Up Protocol

Follow-up consisted of outpatient visits at 3, 6, and 12 months and included a 12-lead ECG at each visit. Seventy-two-hour Holter monitoring was performed at 3 and 12 months; symptom-driven ECGs or event-recorder assessments supplemented the monitoring strategy. None of the included patients had an implantable rhythm-monitoring device (e.g., pacemaker, CIED, or implantable cardiac monitor); therefore, continuous monitoring was not available, and device interrogations were not performed. Arrhythmia recurrence was defined as any atrial tachyarrhythmia (AF, atrial flutter, or atrial tachycardia) lasting ≥ 30 s after a 90-day blanking period. Asymptomatic recurrences detected on scheduled monitoring were included.

### 3.5. Outcome Measures

The primary endpoint was freedom from any atrial tachyarrhythmia ≥ 30 s off antiarrhythmic drugs (AADs) after the 90-day blanking period up to 12 months of follow-up. Per the study SOP, AADs were discontinued for all patients in both groups at the end of the 90-day blanking period and were only re-introduced if a recurrent atrial tachyarrhythmia occurred. Secondary endpoints included procedure duration, fluoroscopy time, number and distribution of reconnected PVs, periprocedural complications (including phrenic nerve palsy), length of hospital stay, and AAD use at 12 months.

### 3.6. Statistical Analysis

Continuous variables are reported as mean ± SD or median (IQR), as appropriate. Between-group comparisons: Student’s *t*-test or Mann–Whitney U-test. Categorical variables: Fisher’s exact test. Effect sizes with 95% CIs are reported for key differences (e.g., mean number of reconnected PVs, procedure time). A multivariable Cox proportional hazards model was constructed to adjust for age, left atrial size, AF type (paroxysmal vs. persistent), and time from index ablation to redo. A two-sided *p* < 0.05 was considered significant. Effect sizes were reported with 95% Cis.

## 4. Results

### 4.1. Baseline Characteristics

Sixty-one patients (41 men, 20 women) with a mean age of 66 ± 10 years were included: 32 in the CB Redo group and 29 in the RF Redo group. Coronary heart disease was present in 30 patients (50%): 19 in the CB Redo group and 11 in the RF Redo group. Mean left ventricular ejection fraction was 56% vs. 55% across groups. All patients had arterial hypertension. Mean left atrial volume was 63 ± 13 mL in CB Redo and 62 ± 6 mL in RF Redo; *p* = 0.9 (Table 1).

### 4.2. Arrhythmia at Recurrence

**Figure 3:**
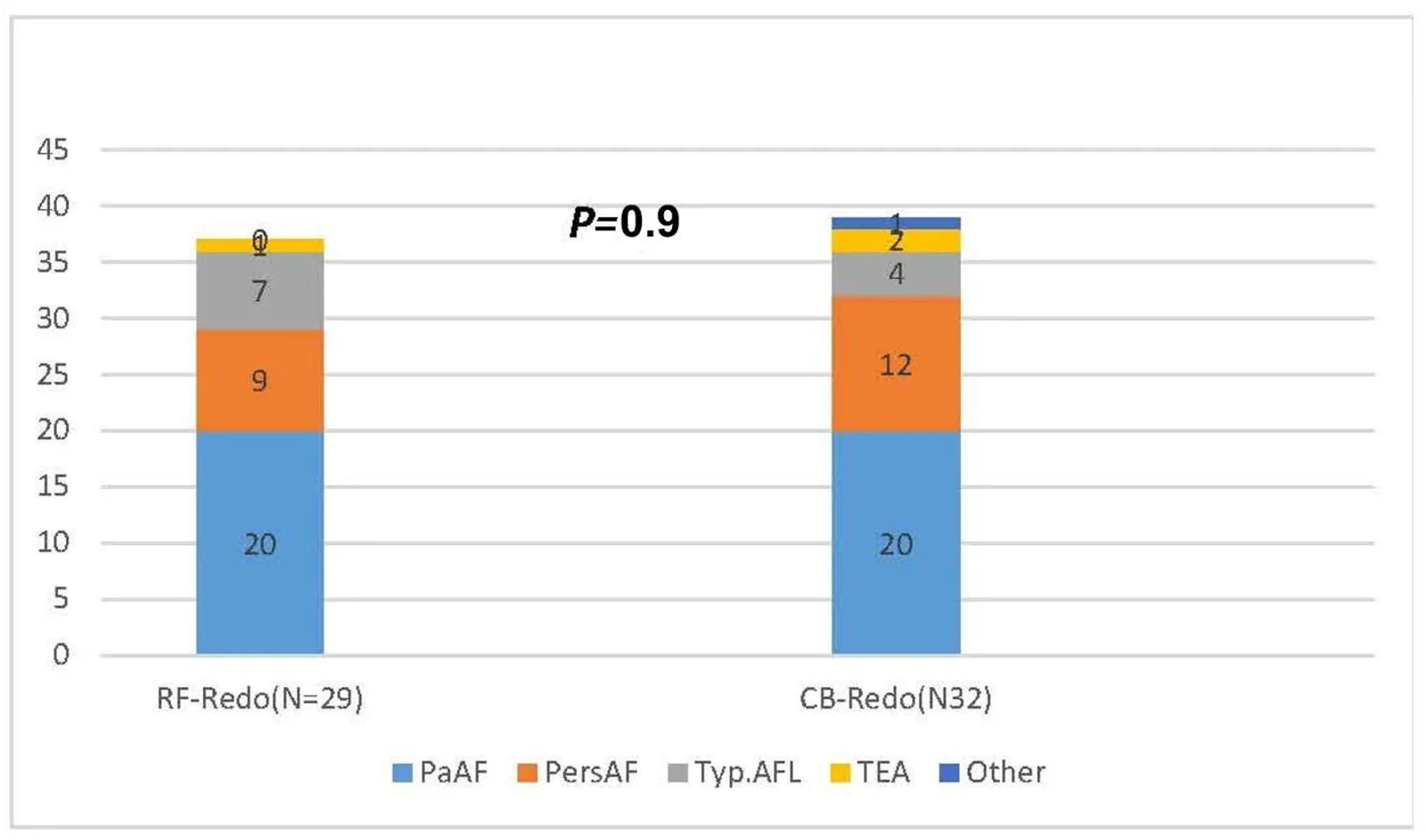
Recurrent arrhythmias before redo-ablation: Paroxysmal atrial fibrillation (AF) was the most prevalent recurrent arrhythmia (65%, 40 patients) in the collective of all patients (69% in the radiofrequency group and 62% in the cryoballoon group). Nine patients had persistent atrial fibrillation (31%) in the RF group and 12 (37.5%) in the CB group. Seven patients (24%) in the RF group and 4 (12%) in the CB group had atrial flutter. There were 3 (9%) other atrial tachycardias in the CB cohort (1 in RF-redo versus 2 in the CB-redo).

Overall, recurrent arrhythmia before redo ablation was paroxysmal AF in 65% and persistent AF in 34% of patients. Paroxysmal recurrence was numerically more frequent in the RF group (69%) than in the CB group (62%) (Figure 3).

### 4.3. Procedural Timing and PV Reconnection

**Figure 4:**
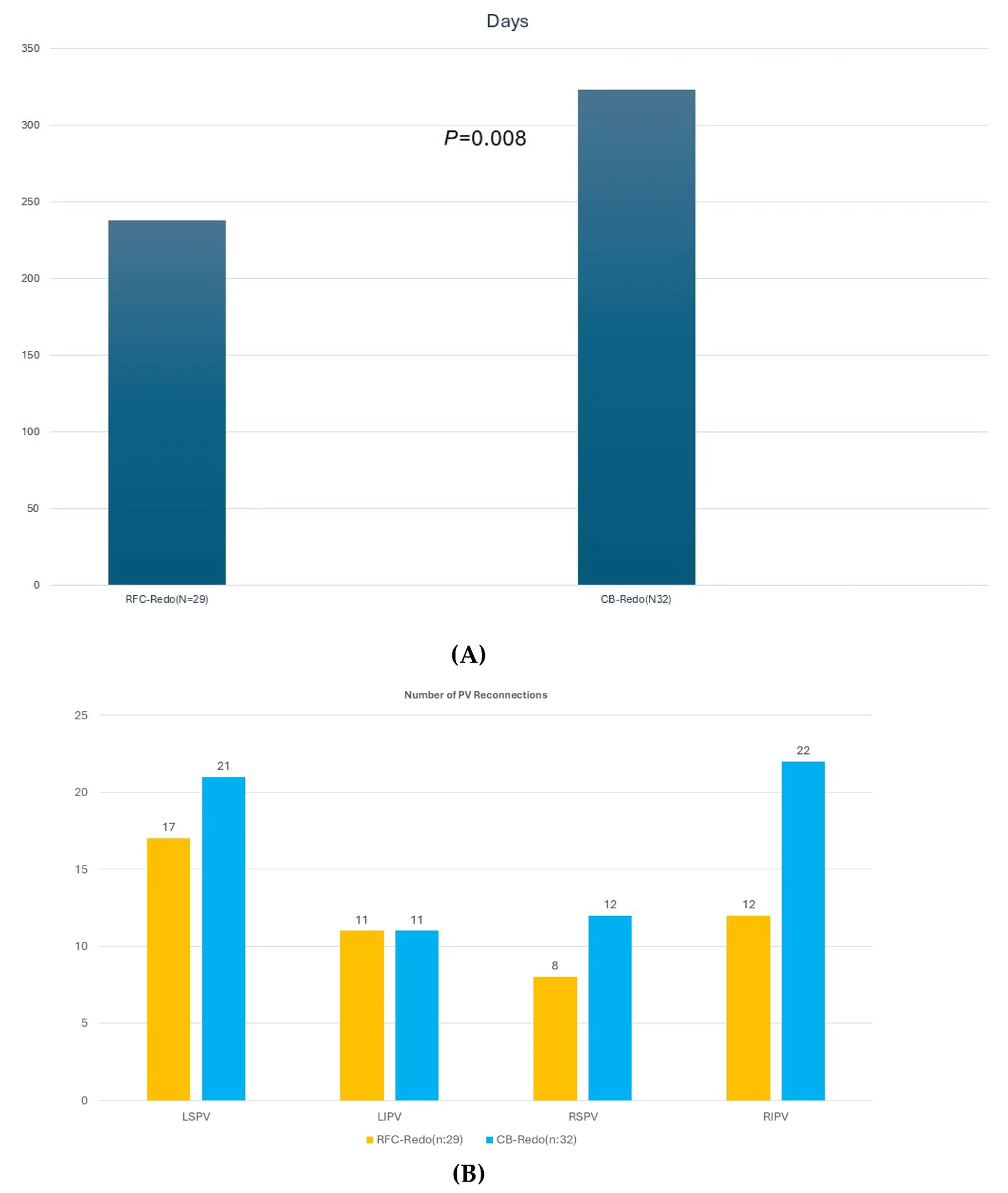
(**A**) The mean time between index procedure and redo ablation was significantly different between the cryoballoon redo group and the radiofrequency redo group (323 and 238 days, *p* = 0,008). (**B**) Number of Pulmonary vein (PV) reconnections during repeat ablation. In the CB group, the number of reconnected PVs was not significantly higher than in the RF group (*p* = 0.08), but it was significantly higher for RIVP (*p* = 0.03).

The median interval from index CB ablation to redo procedure differed significantly between the RF and CB groups (238 days vs. 323 days; *p* = 0.0008). At redo, all patients had at least one reconnected PV. The mean number of reconnected PVs per patient was numerically higher in the CB group (2.1 ± 1.1) than in the RF group (1.7 ± 0.6). Reconnection rates were numerically higher in CB-Redo for LSPV (65,6% vs. 58%; *p* = 0.77), RSPV (37% vs. 27%; *p* = 0.41), and significantly higher for RIPV (69% vs. 41%; *p* = 0.03) but not for LIPV (34% vs. 38%; *p* = 0.77) (Figure 4A). The number of reconnected PVs per patient in both patient cohorts is depicted in Figure 4B.

### 4.4. Procedural Characteristics, Complications, and Length of Stay

CB procedures had a significantly shorter mean total procedure duration than RF. Fluoroscopy time did not differ significantly between groups. No major periprocedural complications were observed in either cohort, including phrenic nerve injury. The median post-procedural hospitalization was two days in both groups.

### 4.5. Antiarrhythmic Therapy and Early Outcomes

Antiarrhythmic drug regimens post-reablation were similar, although amiodarone use was numerically higher in the RF group (14/29, 48%) than in the CB group (11/32, 34%; *p* = 0.30). Early recurrences during the blanking period were numerically more frequent in the RF cohort (9/29, 31%) than in CB (8/32, 25%) and were managed with electrical cardioversion. Overall freedom from any atrial tachycardia ≥ 30 s during follow-up was 79% across the cohort, with no significant difference between redo modalities (recurrence after CB redo: seven patients (21.9%); and six (20.7%) patients after RF redo; *p* = 0.75).

### 4.6. Time-to-Event Analysis and Multivariable Modeling

**Figure 5:**
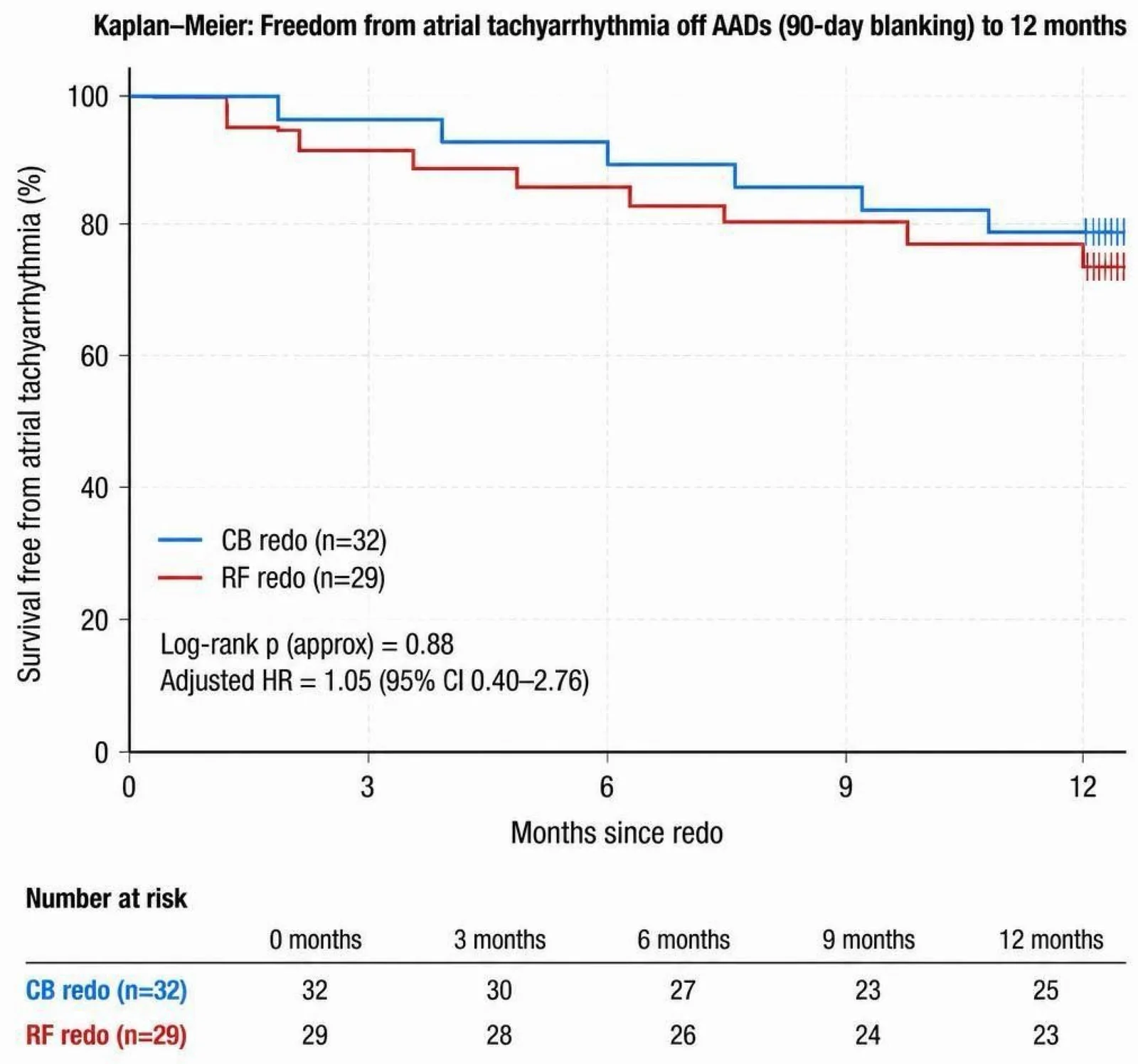
Kaplan–Meier curves for freedom from atrial tachyarrhythmia in both groups.

Kaplan–Meier curves for freedom from atrial tachyarrhythmia are shown in Figure 5. Log-rank *p* = 0.88. A multivariable Cox proportional hazards model adjusted for age, left atrial size, AF type, and time from index to redo yielded an adjusted HR (CB vs. RF) = 1.05 (95% CI 0.40–2.76; *p* = 0.92). Total first-recurrence events included = 13. Events-per-variable in the Cox model were limited; the adjusted estimates should therefore be interpreted cautiously.

## 5. Discussion

Atrial fibrillation (AF) is the most common arrhythmia, increasing with age and morbidity. One important aspect of diagnosis is thromboembolic risk stratification [9]. Recent guidelines for AF management indicate that AF ablation is a Class IIa recommendation with level-B evidence in selected patients with symptomatic AF, and a Class I recommendation after one failed or intolerant class I or III AAD to improve symptoms of AF recurrences in patients with AF [10]. Early rhythm control therapy using ablation, was associated with a lower risk of adverse cardiovascular outcomes than usual care among patients with early atrial fibrillation and cardiovascular conditions [11]. Late treatment of AF with catheter ablation has been reported to affect procedural success. For example, Bunch et al. reported that a later ablation after diagnosis was associated with a significant negative impact on procedural success and AF recurrence in delayed catheter ablation [12].

If patients with paroxysmal AF are not treated, an annual progression risk of up to 15% from paroxysmal AF to persistent AF is reported [13]. At five years, approximately 25% of patients with persistent atrial fibrillation progress to long-standing persistent or permanent AF [14]. This disease progression may cause both structural and electrical atrial remodeling, which may affect long-term treatment outcomes. The two interventional therapeutic approaches, radiofrequency ablation and cryoballoon ablation, have proven very effective in treating atrial fibrillation. The question is which therapeutic approach, cryoballoon ablation or radiofrequency ablation, is more effective as a redo procedure after the recurrence of atrial fibrillation after initial pulmonary vein isolation (PVI) using a cryoballoon. We have addressed this question in the present study.

In the present study, cryoballoon ablation had a significantly shorter total procedure duration than the radiofrequency procedure. Of note, RF ablation is performed using point-by-point circumferential ablation to surround the pulmonary veins, which may explain this result. The fluoroscopy time for cryoballoon ablation is typically longer than that for radiofrequency ablation; however, in this study, the fluoroscopy time was similar between the groups.

In this comparative study, no complications were documented. Even phrenic nerve injury, one of the principal complications of cryoballoon ablation, was not detected peri- or post-procedurally. Notably, phrenic nerve injury is generally reversible and has been reported in up to 4.2% of cryoablation procedures [15,16].

Pulmonary vein isolation using radiofrequency energy with point-by-point ablation creates circumferential isolation of the pulmonary vein and, therefore, a larger area of atrial lesions. This broad area of the lesions can provide a substrate for atrial flutter or other arrhythmias [17]; however, in our study, the risk of atrial flutter did not differ significantly between the cohorts.

In contrast to cryoballoon ablation, RF ablation offers high-density endocardial mapping and three-dimensional reconstruction of the left atrium. This helps identify low-voltage areas, fragmented activity, ectopic foci, and rotors [18]. In our study, RF had no additional benefit in preventing recurrence compared with cryoballoon ablation; however, it significantly extended treatment duration (by approximately one hour) and fluoroscopy time (by approximately 12 min) [19].

We used contact force sensing RF catheters for all RF-redo procedures and guided point-by-point lesion delivery using contemporaneous lesion-quality metrics available at the time. Specifically, operators targeted a contact force of 10–20 g per lesion, monitored Force–Time Integral (FTI) as the primary lesion effort metric, and used impedance drop and delivered power/time as adjunctive indicators of lesion formation. When available, Lesion Size Index (LSI) values were recorded and used as a secondary guide for anterior versus posterior targets; however, FTI and impedance dynamics were the dominant indices informing consolidation and additional touch-up lesions. These parameters directed decisions about lesion spacing, need for repeat applications, and the choice between focal RF touch-ups versus broader circumferential re-isolation. The use of the contact force metrics likely influenced lesion durability and reconnection patterns observed in the RF cohort and should be considered when comparing outcomes with the CB-redo group [20]. 

An alternative for redo procedures after index cryoballoon ablation is pulsed field ablation (PFA); recent data support its safety and efficacy in this setting [21]. PFA may offer faster, myocardial-selective lesions and lower collateral risk, and should be considered when available.

Changes to the index strategy can influence redo energy choice. Focal PV reconnection after cryoballoon ablation often favors targeted radiofrequency ablation, while diffuse reconnection or challenging anatomy may prompt repeat balloon-based ablation or PFA. Operator experience, lesion pattern at redo, and local algorithmic criteria should guide the decision.

Notably, the 3D-mediated proportion of fibrosis in the RF group may serve as a surrogate marker of advanced atrial remodeling and as a negative predictor of unsuccessful re-ablation. Evaluation of fibrosis after cryoballoon ablation is not feasible.

In the present study, ablation procedures in both groups were limited to pulmonary veins alone. The question arises whether the use of additional ablation lesions beyond pulmonary veins, such as low-voltage areas, lines, fragmented activity, ectopic foci, and rotors, may affect the outcome. Apparent freedom from AF after ablation does not eliminate stroke risk, particularly with intermittent rhythm surveillance. An updated meta-analysis of 267,443 post-ablation patients [22] evaluated thromboembolic and bleeding outcomes associated with anticoagulation continuation vs. discontinuation. Although informative, the observational nature and selection biases limit causal inference; anticoagulation decisions should remain individualized based on thromboembolic and bleeding risk assessment.

This study did not assess quality of life, symptom relief, return to activities, or patient satisfaction. These patient-centered outcomes may differ by energy source and should be incorporated in future comparative redo-ablation studies [23].

Artificial intelligence for strategy selection: AI-assisted models integrating imaging, procedural biophysics, electroanatomic maps, and clinical variables may aid in the selection of redo strategy and predict reconnection patterns. These methods require robust external validation and demonstration of clinical utility before routine adoption [24].

## 6. Conclusions

This analysis revealed an overall success rate of 79% in patients with recurrent atrial fibrillation who underwent redo treatment with either radiofrequency ablation or cryoballoon ablation. Operators should choose between CB ablation and RF ablation in redo ablation based on patient characteristics and features, while taking into account the operator’s experience and preference.

## 7. Clinical Interpretation and Limitations

Although point estimates are similar, the limited sample size and small number of events produce wide confidence intervals and low precision; therefore, equivalence cannot be inferred. Treatment selection was non-random and operator-driven, which may bias comparisons. Monitoring was intermittent; asymptomatic recurrences may have been missed. The Cox model included several covariates with limited events; results should be interpreted cautiously.

## Figures and Tables

**Table 1:** Baseline Patient Demographics and Comorbidities for Repeat Ablation.

Characteristic	RF Redo (n:29)	CB Redo (n:32)	*p*-Value
**Age, years**	63.4 ± 11	69 ± 11	0.45
**Sex (M/W)**	20/9	21/11	0.78
**Paroxysmal**	20	20	0.28
**Persistent**	9	12	0.98
**Atrial flutter**	7	4	0.32
**transarterial embolization**	1	2	0.6
**CHA2DS2-VASc-Score**	2.2 ± 1.2	2.8 ± 1	0.08
**Procedure time, hours**	2:08 h	1 h	0.04
**Time between initial ablation and redo, ms**	238 ± 48	323 ± 72	0.0008
**Number of PV Reconnections**	50	69	0.08
**Mean number of reconnected PVs**	1.7 ± 0.6	2.1 ± 1.1	0.08
**Recurrence in the Blanking time**	9	8	0.6
**Number of cardioversions**	1.3	1.5	0.6
**European Heart Rhythm Association classification**	2.5	2.4	0.5
**Coronary heart disease**	11	19	0.2
**Mild mitral valve insufficiency**	17	27	0.08
**Left atrial (LA) Size, mm**	62.4 ± 6	62.5 ± 13	0.9
**Ejection fraction (EF), %**	56%	55%	0.6
**Diastolic dysfunction**	29	32	1.00
**Chronic obstructive pulmonary disease**	3	5	0.7
**Drug treatment at discharge**			
**Amiodarone**	14	11	0.3
**Other antiarrhythmic (AA)**	0	0	-
**ß-Blocker**	27	30	0.9
**ACE/AT1**	18	20	0.9

## Data Availability

The original contributions presented in this study are included in the article. Further inquiries can be directed to the corresponding authors.
